# Practical Remarks on Some Exhausting Diseases

**Published:** 1845-07

**Authors:** 


**Practical Remarks on some Exhausting Diseases, particularly those Incident to Women.** By Sir *James Eyre*, M.D. Physician to the St. George's and St. James's Dispensary. 8vo. pp. 75. Churchill, 1845.

The chief object of this little modest brochure is to extend the use of a comparatively modern remedy to many complaints for which it has not hitherto been prescribed. The readers of this Journal are aware that, twenty or thirty years ago, the nitrate of silver was seldom exhibited except in obstinate cases of epilepsy—and that with a trembling hand and sceptical mind, lest the patient should turn blue and still retain the malady! This impression greatly limited the employment of the remedy which, however, gradually extended to a number of gastric affections rebellious to other medicines. It is only a very few years since another preparation of silver was introduced into practice recommended as not likely, or not capable of tinging the skin—viz: the oxyde of silver. This has been exhibited with more freedom, and with less fear. But we may state, en passant, that we have seen a case where even the oxyde has slightly tinged the skin of a delicate female who had taken the preparation three or four months. It is rarely necessary, however, that such protracted exhibitions should be employed, except in epilepsy, the generality of other complaints only requiring the remedy for a few weeks.

The author, who is a shrewd and observant practitioner, and has opportunities very nearly as great as the physicians of metropolitan hospitals have, for testing the efficacy of remedies, has furnished his professional brethren with a very plain and unpretending record of cases treated at the Dispensary—chiefly pyrosis, gastric disorder, slowly exhausting hæmorrhage from mucous surfaces; "but above all, atonic hæmorrhagia, which, though arising from various causes, and hence often perplexing to the practitioner, will, it is confidently predicted, become henceforth, as amenable to treatment as it has been hitherto unmanageable." After referring to preceding writers—chiefly Lane, Serre, Golding Bird, Clendinning, &c. Sir James proceeds to pyrosis, pronounced by Baillie to be one of the *Opprobria Medicorum.*

"Whatever opinion be the correct one, the pathological conditions being so obscure, it will be found, that however it may be induced, the oxyde of silver given in half-grain doses thrice daily, will prove more effectual in its cure than any medicine which has yet been employed. That it has not once failed in the author's hands, let the following illustrative cases, a few of many, bear witness; all the patients were women. It should be premised, however, as costiveness generally existed, that one or two of Dr. Hamilton's pills (of Edinburgh) composed of Ext. coloc. c. ℈ij., Ext. hyoscyami ℈j., divided into xii., were given every night." 6.

Ten cases are detailed illustrative of the efficacy of the remedy, one or two of which we shall glance at.

*Case* 1.—M. B. aged 30, married, had suffered from pyrosis for sixteen years. No tenderness of epigastrium on pressure. A quarter of a grain of the oxyde of silver thrice a day, with proper dietetic instructions. In less than a fortnight the symptoms were removed; but she relapsed on eating improper food. A return to the oxyde soon produced ease, and she continued well.

*Case* 2.—" E. D. aged 23, single; sempstress. Sept. 14th, 1843. Had been ill two months, bringing up a pint or more daily, of a tasteless watery fluid; could not bear pressure; ordered to apply a dozen leeches to the pit of the stomach, and the bowels being confined, to take the colocynth pills. On the 18th, the tenderness having subsided, began with the oxyde. 21st, nearly well. Oct. 2nd. Still improving: desired to try the following tonic mixture: ℞ Acidi hydrochl. d. ʒj., Inf. chirayitæ ℥viiss., Tinct. card. c. ℥ss. Ft. mist. Coch duo ampla ter quotidie. Nov. 1st, slight return of pyrosis: directed to resume the powders, which in eight days removed all her ailments; and in Sept. 1844, she called, and stated that, during the past year, there had been no return of her complaint." 10.

*Case* 3.—M. S. ætat. 41, married, Nov. 20, 1844. Had suffered great pain in her stomach for six years, which pain had lasted, at one time, for a year and a half without intermission. Bowels open—no epigastric tenderness—frequent inclination to vomit. Was desired to restrict herself as much as possible to a milk diet. In six days she was so much relieved by half-grain doses of the oxyde thrice a day, that, to use her own expression, " she felt herself quite another person." By the 31st Dec. all medicine was left off, she being well.

Our author next adverts to the remedy in hæmoptysis and hæmatemesis. Passing over some ingenious theoretical and practical observations, we shall allude to a few cases of hæmoptysis in which the oxyde of silver was employed.

*Case* 1.—J. T. aged 22, in the Kent-road, applied on the 9th March, 1842, having had occasional attacks of spitting of blood for eighteen months past. He had cough—emaciation—no rigors—pulse 85 and weak—bronchial respiration, with mucous râle—dulness under the right clavicle—no hereditary disposition to phthisis. Hæmoptysis, more or less, had now continued regularly for three months. He was ordered to keep very quiet, and to take one grain of the oxyde, with ten grains of the comp. powder of tragacanth ter die. " The bloody discharge, from this time, gradually diminished, and on the 19th April, he had gained flesh and looked much better." His cough was greatly relieved—appetite good—sleep refreshing. Was ordered quinine and sulphuric acid, with gentian. From this time he ceased to attend the Dispensary.

After detailing several cases of hæmatemesis and hæmoptysis, our author concludes thus:—

" On a review of these few cases, whether of hæmoptysis or hæmatemesis, enough has been detailed to evince the great power of our remedy, and, although the author is not so insane as to profess to cure tubercular phthisis, he may be considered, perhaps, to have been useful in his generation, by the endeavour to push into light an additional means of arresting pulmonary hæmorrhage, though only for a time, and thereby relieving the pitiable distress of patients and their friends, caused by this dreaded symptom; and, though the good that we do be not permanent, we may yet, on the score of humanity, perchance, propitiate that eminent Parisian physician, M. Velpeau, who, in an 'Essai sur les Tubercles,' published, not many years ago, in the French metropolis, says, 'Angli ipsi superbi, illi æmulatores, qui se nobis præstare semper contendunt, quique nunquam vel pares esse potuerunt, ne quidam duas ideas inter se cohærentes, hâc de questione ediderunt.'" 31.

*Uterine Hæmorrhage.*—Our author glances most rapidly at those means which have hitherto been considered useful in restraining this species of the profluvium, when it continues for weeks or months after parturition—after menstruation, and under other circumstances. These opinions and authorities we need not review. A series of thirty-one cases of mænorrhagia is briefly detailed by the author, of which we can only take one, without any selection.

*Case.*—"J. M., aged forty. April 3rd, 1842. Married seventeen years; no family; the catamenia, which were always regular, and never profuse, had first appeared at the age of twelve. Had now been afflicted for three months, in an extreme degree, and the discharge had been unusually profuse the day before she applied for relief; her pulse was feeble; there was no bearing down, nor pain. That the flow under these circumstances might be checked gradually, the minimum dose of the medicine was prescribed: after two days there was an amendment; in a week the hæmorrhage, for such it was, had nearly ceased, and in another, entirely disappeared. The patient having remained free of all complaint till the end of May, was then dismissed cured." 45.

The following is the concluding passage of this little practical brochure.

"This, then, concludes the detail of the majority of those instances, in which the oxyde of silver has been found so superior to all other means employed by the writer during an active professional life of more than thirty years; and he feels himself fully justified in inviting others to test a medicine, by the success of which, their usefulness may be increased, and the care which he has taken in arranging and presenting his cases, more than amply rewarded. That it is a tonic, and a sedative, as Dr. Golding Bird believed in 1840, and 1841, there can be no doubt; that it is a safe, and efficient astringent, there is good evidence to prove; the property of a specific is not assigned to it, for all know how very few of our remedial agents possess that character.

"Every experienced physician will admit that, owing to idiosyncracy and diversity of constitution, the same disorder presents a different aspect in almost every patient, and hence, that each case must be individually studied, and our remedial measures varied, in order to meet its peculiarities. John Abernethy, of glorious memory, (who taught his pupils to think for themselves, and not to submit, without inquiry, to any man's dictum,) was accustomed to say to them, quaintly and characteristically, 'Gentlemen, there is no such thing as a remedy for a disease, excepting sulphur, for a complaint which is never alluded to in good society, and (perhaps) mercury in another disorder, of at least, equally bad repute.'

"It will have been observed that the dose of the oxyde of silver employed by the writer in the foregoing cases never exceeded *three* grains a day, instead of six, as given on its first introduction, and that its employment is not recommended where febrile action exists in any considerable degree. In addition to its value in gastrodynia, in pyrosis, in hæmoptysis, in hæmatemesis, and in the first and second classes of menorrhagia, of Dr. Fleetwood Churchill, it will be found to be productive of infinite benefit in restraining, when absolutely necessary, hæmorrhage proceeding from the intestinal canal, obstinate chronic diarrhœa, colliquative perspirations, leucorrhœa, and other maladies, in the treatment of which, the writer is at the present time, extensively testing its efficacy." 75.

The thanks of the profession are due to Sir James Eyre for this plain and succinct detail of important cases treated by a safe and easy remedy which is only just coming into use.

